# Evaluating thyroid hormone disruption: investigations of long-term neurodevelopmental effects in rats after perinatal exposure to perfluorohexane sulfonate (PFHxS)

**DOI:** 10.1038/s41598-020-59354-z

**Published:** 2020-02-14

**Authors:** Louise Ramhøj, Ulla Hass, Mary E. Gilbert, Carmen Wood, Terje Svingen, Diana Usai, Anne Marie Vinggaard, Karen Mandrup, Marta Axelstad

**Affiliations:** 1 Division of Diet, Disease Prevention and Toxicology, National Food Institute, Technical University of Denmark, Kgs. Lyngby, DK-2800, Denmark; 2 Center for Public Health and Environmental Assessment, Office of Research and Development, U.S. Environmental Protection Agency, Research Triangle Park, North Carolina, USA

**Keywords:** Thyroid hormones, Endocrine system and metabolic diseases, Development of the nervous system

## Abstract

Thyroid hormones are critical for mammalian brain development. Thus, chemicals that can affect thyroid hormone signaling during pregnancy are of great concern. Perfluorohexane sulfonate (PFHxS) is a widespread environmental contaminant found in human serum, breastmilk, and other tissues, capable of lowering serum thyroxine (T4) in rats. Here, we investigated its effects on the thyroid system and neurodevelopment following maternal exposure from early gestation through lactation (0.05, 5 or 25 mg/kg/day PFHxS), alone or in combination with a mixture of 12 environmentally relevant endocrine disrupting compounds (EDmix). PFHxS lowered thyroid hormone levels in both dams and offspring in a dose-dependent manner, but did not change TSH levels, weight, histology, or expression of marker genes of the thyroid gland. No evidence of thyroid hormone-mediated neurobehavioral disruption in offspring was observed. Since human brain development appear very sensitive to low T4 levels, we maintain that PFHxS is of potential concern to human health. It is our view that current rodent models are not sufficiently sensitive to detect adverse neurodevelopmental effects of maternal and perinatal hypothyroxinemia and that we need to develop more sensitive brain-based markers or measurable metrics of thyroid hormone-dependent perturbations in brain development.

## Introduction

Thyroid hormones are critical for normal brain development. The spatiotemporal action of thyroid hormones is essential for orchestrating the developmental processes of neurogenesis, migration, synaptogenesis, and myelination^1,2^. In humans, insufficient thyroid hormone levels at critical times in neurodevelopment can induce long-term intellectual and behavioral impairments^3,4^. These outcomes can have serious consequences for both the affected individuals and society as a whole, including increases in healthcare expenses and a reduction in lifetime income^5,6^.

The thyroid hormone system can be affected by insufficient iodide intakes, exogenous substances such as chemicals and drugs, or by some diseases. Congenital hypothyroidism and iodine deficiency, conditions that severely reduce thyroid hormones during early development, result in severe mental retardation. However, even subclinical maternal T4 deficiency (hypothyroxinemia) can impact neurodevelopment and IQ of the child^7–12^.

In rodents, severe thyroid hormone deprivation clearly impairs brain development^13–20^. Yet, with regard to low-grade thyroid hormone disruption, a relationship with adverse effects on brain development is less obvious^21–27^. Still, environmental chemicals can perturb the thyroid axis through a variety of mechanisms, often inducing more subtle changes in hormonal status than seen with anti-thyroid drugs such as propylthiouracil (PTU) and methimazole (MMI)^3,4,28,29^. This means that, to better protect humans against thyroid disruption, we need to allow for more sensitive assays in rodent toxicity testing. This, because establishing a relationship between modest degrees of serum thyroid hormone decrements and neurodevelopmental sequelae is crucial for the regulation of thyroid disrupting chemicals, as current legislative regulation of thyroid disruptors relies on linking a change in serum hormone levels to a consequential adverse effect.

**Figure 1:**
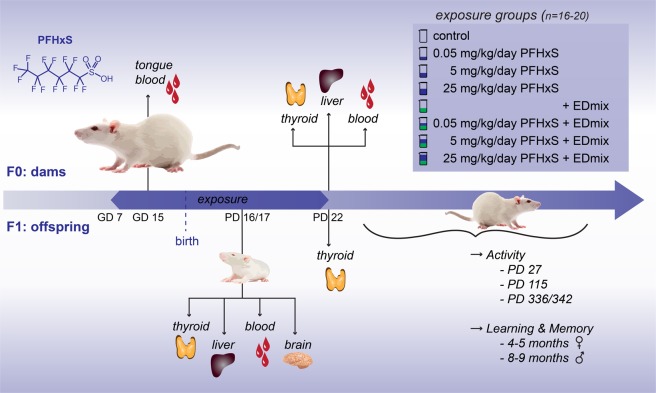
Overview of the study. Time-mated rat dams were exposed to PFHxS with or without a mixture of environmentally relevant endocrine disrupting chemicals from gestation day 7 through to postnatal day 22. Blood was taken from dams and pups for thyroid hormone assessments and organs were excised, weighed and stored for further analysis on PD 16/17 and 22. A subset of offspring was weaned (PD 22) and used for testing of motor activity levels at three ages, and learning and memory in the radial arm maze (4–5 months of age for the females and 8–9 months for the males). The litter was used as statistical unit for all analyses. PFHxS: Perfluorohexane sulfonate, GD: Gestation day, PD: Postnatal day.

Poly- and perfluoroalkyl substances (PFAS) constitute a class of environmental chemicals that is frequently reported to cause thyroid hormone disruption^30^. PFHxS is found in drinking water supplies^31^ and has repeatedly been one of the top three PFASs to which humans are environmentally exposed^32–34^, yet its toxicological effects remain poorly characterized^35–37^. We recently reported that developmental exposure to perfluorohexane sulfonate (PFHxS) caused a marked reduction in total serum thyroxine (T4) levels in both rat dams and their offspring^36^. Herein, to address the possible health impacts of PFHxS exposure, we investigated the effects on the thyroid hormone system and potential neurobehavioral consequences (see Fig. 1). Additionally, we examined the effects of PFHxS with concurrent exposure to a mixture of 12 common endocrine disrupting chemicals, denoted EDmix, to mimic a more realistic exposure scenario as it pertains to humans^38,39^.

## Results

### Thyroid hormones in dams and offspring

**Figure 2:**
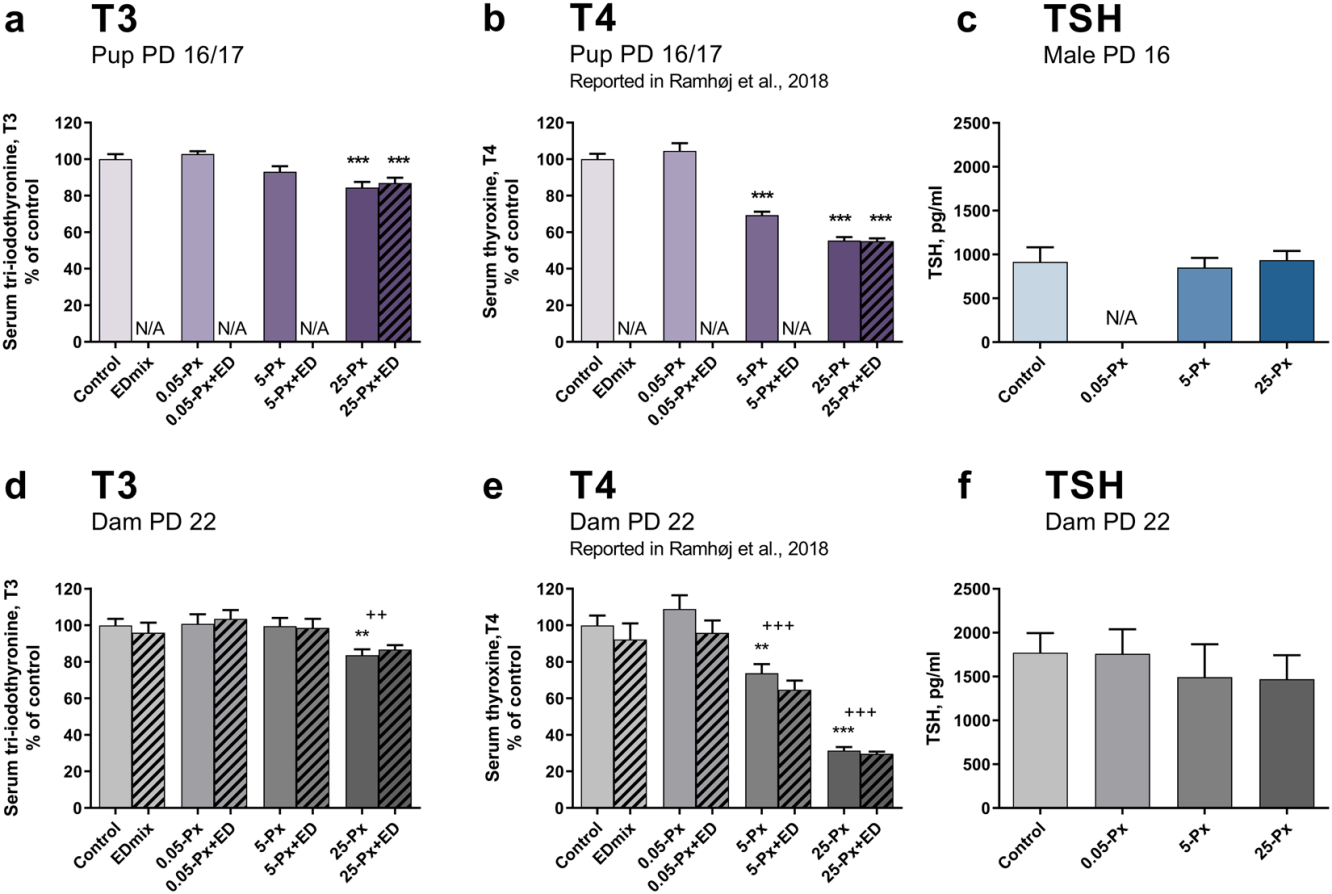
T3, T4 and TSH after developmental exposure to PFHxS. (**a**) PFHxS decreased pup T3 levels at 25 mg/kg on PD 16/17, mean of the controls was 1.48 nM. Data shown as mean + SEM. n = 14–16 (except control with n = 18) litters represented by either a male or a female pup. (**b**) Pup T4 was reduced from 5 mg/kg PFHxS (absolute values previously reported^36^. n = 14–16 (except control with n = 18) litters represented by either a male or a female pup. (**c**) TSH in male pups PD 16. n = 11–13. (**d**) T3 levels in dams on PD 22, mean of the controls was 1.45 nM. n = 13–15. (**e**) Dam serum T4 at PD 22 was reduced from 5 mg/kg PFHxS (absolute values previously reported^36^). n = 13–15 (except control with n = 20). (**f**) Dam serum TSH at PD 22. n = 15–16. Data shown as mean + SEM. **p < 0.01 compared to control, ***p < 0.001 compared to control, ^++^p < 0.01 for full model comparison of indicated dose of PFHxS compared to no PFHxS exposure in the control and EDmix group. ^+++^p < 0.001 for full model comparison of indicated dose of PFHxS compared to no PFHxS exposure in the control and EDmix group. ED: EDmix. Px: PFHxS. PD: postnatal day. TSH: Thyroid stimulating hormone. T3: Tri-iodothyronine, N/A: not available.

The dose-dependent reductions in total serum T4 levels in both pups and dams after developmental exposure to PFHxS were previously reported^36^. For completeness, some of these data are included here as percent of control (Fig. 2b,e), to support a full appreciation of the impact of PFHxS on the thyroid hormone signaling axis. Herein, we show T3 and TSH determinations to complement the characterization of the thyroid system after PFHxS exposure.

#### Dams

As reported previously, maternal T4 levels on GD 15 were significantly decreased to 80 and 60% of control levels by 5 and 25 mg/kg PFHxS, respectively^36^. The effect on T4 was even more pronounced on PD 22, when levels were down to 70 and 30% of controls, respectively (Fig. 2e)^36^. Effects on dam T3 levels were less marked, but statistically significant decreases were observed. On GD15, dam T3 levels were slightly reduced to 97% at 25 mg/kg PFHxS, (p = 0.0024, full model, data not shown). A greater reduction to 84% of controls was seen on PD 22 in the 25 mg/kg PFHxS group (p = 0.0099 in the simple model and p = 0.0054 using the full model, Fig. 2d). TSH in dams was measured on PD 22 and was not altered by exposure to PFHxS (Fig. 2f).

#### Offspring

Pup T4 was affected on PD 16/17, with reductions to 70 and 55% of control levels in the 5 and 25 mg/kg group, respectively (Fig. 2)^36^. Pup T3 levels were also reduced by PFHxS exposure as significant 7–16% reductions in T3 were evident in the 25 mg/kg exposed pups (p = 0.0002 for 25-Px and p = 0.0015 for 25-Px + EDmix. Fig. 2a). As seen in dams, TSH levels were not changed in PD 16/17 pups (Fig. 2c, females not shown). There was no effect of the EDmix on pup T4^36^ and T3 levels (Fig. 2a,b).

### Thyroid gland weight, histopathology and gene expression

#### Dams

On PD 22, no statistically significant effects on dam thyroid gland weights were seen, apart from a slight reduction in response to the EDmix (p = 0.0133, full model, data not shown). Thyroid gland histopathology was not significantly affected by PFHxS exposure, and neither were the expression levels of six gene transcripts involved in thyroid hormone synthesis and regulation (*Slc5a5*(NIS)*, Nkx2.1, Tpo, Tshr, Pax8* and *Dio1*)(data not shown). Thus, TSH levels, thyroid gland weight, histopathology and gene expression indicate a lack of thyroid gland perturbation in response to exposure or as a compensatory reaction to decreases in serum T4.

#### Offspring

**Figure 3:**
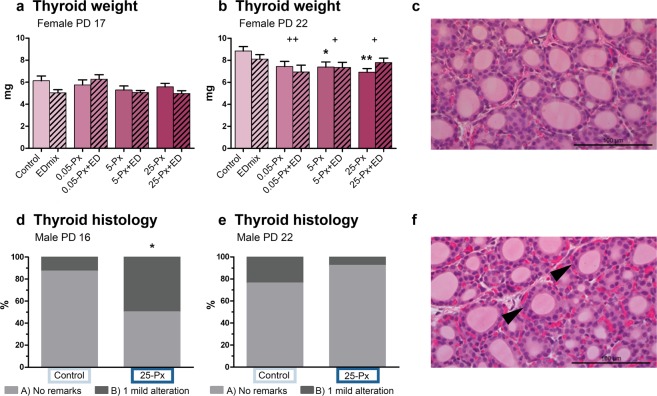
Thyroid gland weight and histology PD 16/17 and 22 after developmental exposure to PFHxS. (**a**,**b**) Female pup thyroid gland weight at PD 17 and PD 22. Data shown as mean + SEM. n = 11–16. (**d**,**e**) Thyroid histopathology on male pup thyroid glands PD 16 and PD 22. Bars represent percentage of animals receiving indicated score. control n = 16–17 and 25-Px n = 13–14. (**c**,**f**) Representative images of thyroid tissue from a control male pup (**c**) receiving a score of A (no remarks) and a male pup from the 25-Px group (**f**) receiving a score of B (1 mild alteration, potentially within natural variation) for altered cellularity (arrowheads). * p < 0.05 compared to control, **p < 0.01 compared to control, ^+^p < 0.05 for full model comparison of indicated dose of PFHxS compared to no PFHxS exposure in the control and EDmix group. ^++^p < 0.01 for full model comparison of indicated dose of PFHxS compared to no PFHxS exposure in the control and EDmix group. ED: EDmix. Px: PFHxS. PD: postnatal day.

In the PD 22 female offspring, dose-dependent decrements in thyroid gland weight were observed (Fig. 3b). These decrements were statistically significant from the lowest exposure of 0.05 mg/kg in the full model, and from 5 mg/kg PFHxS in the simple model. Although a similar pattern was observed on PD 17, these slight reductions in gland weight were not statistically significant (Fig. 3a). Control and high dose (25-Px) male thyroid glands from PD 16 were evaluated for histopathological changes associated with thyroid disruption. Small histological changes were found on PD 16 (Fig. 3c–f) with 50% of the exposed animals, versus 12.5% of controls, receiving a B-score reflecting one minor alteration that remained within the normal range. These differences were no longer evident on PD 22. We did not detect hypertrophy or hyperplasia (C-score) at any time point. As seen in dams, and consistent with a general lack of histopathological findings, there were no differences in the expression levels detected in 4 gene transcripts involved in thyroid hormone synthesis and regulation in PD17 female thyroid glands (*Slc5a5* (NIS)*, Nkx2.1, Tpo*, and *Tshr)* (data not shown).

### Liver

We previously reported increased liver weight in both male and female offspring exposed to PFHxS and/or EDmix during development, first evident in females at exposures of 5 mg/kg PFHxS^36^. Here we extended these examinations to histological assessment of liver sections from female offspring (PD 17), but found no morphological changes in liver that could explain the increases in liver weight (data not shown). Minimal midzonal microvesicular vacuolation was observed, but the change was evenly distributed between exposed and control animals. Neither did histological assessment of dam livers (PD 22) reveal any differences between control and high dose animals (25 mg/kg PFHxS) (data not shown).

### Brain development

#### Cerebral cortical gene expression

**Figure 4:**
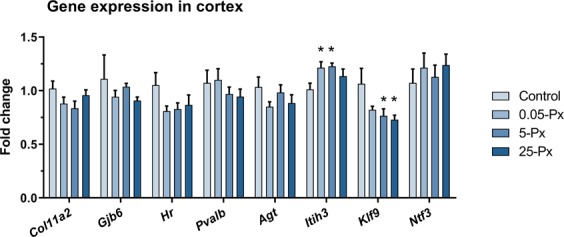
Effects on brain (cortex) gene expression. Cortical gene expression levels for thyroid hormone mediated genes in male offspring PD 16 after developmental exposure to PFHxS. Data shown as mean + SEM. n = 8–9. *p < 0.05 compared to control. ED: EDmix. Px: PFHxS. PD: postnatal day.

The expression of eight gene transcripts was assessed in male offspring on PD 16. These target genes were selected from a suite of genes previously shown to be significantly reduced in the parietal cortex of 14 days old rat pups in response to developmental exposure to the antithyroid drug, PTU^40^. No clear evidence for altered expression due to thyroid hormone insufficiency was observed for *Agt, Col11a2, Gjb6, Hr, Ntf3*, or *Pvalb* (Fig. 4). Expression levels of *Itih3* were slightly increased, which was opposite the effect observed with PTU^40^; however, the direct thyroid hormone response gene target, *Klf9*, was significantly decreased. While the effect size was small (<0.25-fold) the effect was qualitatively consistent with previous reports^40–44^.

#### Motor activity

Habituation, the expected decline in motor activity over the 30 min test period, was observed in offspring at all ages (Supplementary Fig. 1). Similarly, the expected sexually dimorphic pattern was seen in the adult controls, with higher activity levels in females compared to males. Treatment-related effects on motor activity levels at the three ages were minimal and did not correlate with PFHxS dose or thyroid hormone levels during development. Overall, effects suggest disrupted sexual differentiation of the male and female brain, although the results do not clearly indicate whether this mainly arise from an effect in a specific sex or from altered development of both sexes. The most notable changes at the three ages are described below.

**Figure 5:**
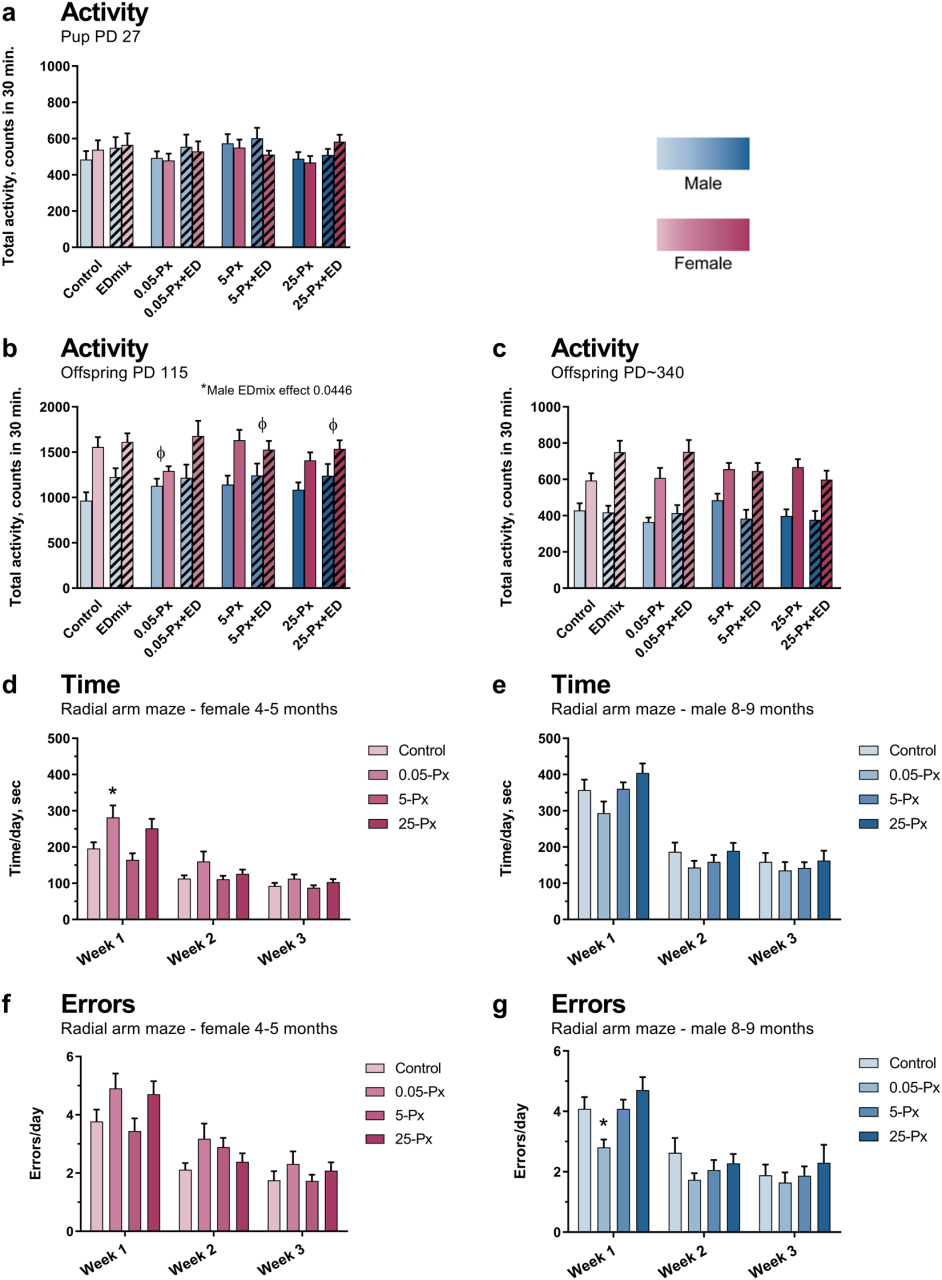
Effects on motor activity levels, and learning and memory after developmental exposure to PFHxS and/or EDmix. (**a–c**) Motor activity levels in offspring PD 27, PD 115 and PD ~ 340. As expected there was an inherent sex difference in activity levels between control males and females in the young adult offspring, this sex difference was not found in the 0.05-Px, 5-Px + ED and 25-Px + ED groups, indicating disturbed sex differentiation of the brain. Pink bars represent female offspring and blue bars male offspring. n = 15–17 (except for EDmix, 0.05-Px + ED and 5-Px + ED with n = 12–13) animals per sex and group. (**d**,**f**) Learning and memory for female offspring assessed in the radial arm maze. n = 16–17. (**e**,**g**) Learning and memory for male offspring assessed in the radial arm maze. n = 17, 15, 19 and 16 for control, 0.05-Px, 5-Px and 25-Px, respectively. Data shown as mean + SEM, *p < 0.05 compared to control, ^ɸ^p < 0.05 for lack of a sex difference within indicated exposure group. ED: EDmix. Px: PFHxS. PD: postnatal day.

In pre-puberty (PD 27) no sexual dimorphism in motor activity levels was observed (as expected for prepubertal rats) and no significant effect of PFHxS exposure was seen in either male or female offspring (Fig. 5a). When dividing the test period into 3 shorter time periods to assess habituation of motor activity, slight but significant increases in activity during the middle period were detected in male offspring exposed to the EDmix (p = 0.014, full model, Supplementary Fig. 1a). No effects were seen in the female offspring (Supplementary Fig. 1b).

In young adulthood (PD 115), no significant effects of PFHxS exposure were seen in either sex, when overall activity count was assessed (Fig. 5b). In contrast, minor reductions in activity were observed in PFHxS females during the middle test period and were limited to the 0.05 mg/kg dose group (Supplementary Fig. 1d). The effects on motor activity were suggestive of disrupted sexual differentiation of the brain. First, the inherent sex difference, which was present in the control group, was not evident in the groups exposed to low-dose PFHxS (both compared to control and in the full model) and in the 5-Px + ED and 25-Px + ED groups (Fig. 5b). Furthermore, exposure to EDmix resulted in an increase in total activity in male offspring, compared to males not exposed to EDmix (p = 0.0446, full model, Fig. 5b), and in the middle testing period (p = 0.0297, full model, Supplementary Fig. 1c), a pattern similar to that observed for EDmix in PD 27 male offspring. These results suggest disrupted sexual differentiation of the brain, limited to the lowest dose of PFHxS, and at higher doses when delivered against a background EDmix exposure.

When the animals were tested for the third time (~PD 340), the expected sexually dimorphic pattern of higher activity levels in females remained(Fig. 5c). Yet, indications that the EDmix affected brain development were persistent. The most notable effect was an increase in activity in the initial test period in EDmix group females when compared to the control group (p = 0.0082, t-test versus control) (Supplementary Fig. 1f). Interestingly, PFHxS seemed to counteract this effect as there was reduced activity in the 25-Px + ED group compared to the EDmix only (Supplementary Fig. 1f).

#### Learning and memory in the radial arm maze

Both male and female offspring showed improved performance during the three-week testing period, manifested as decreased time to complete the test and a reduction of errors made (Fig. 5d–g). While some statistically significant exposure related effects were seen, these were not associated with reductions in serum thyroid hormone levels. The only statistically significant PFHxS exposure-related effects were seen at 0.05 mg/kg PFHxS, where males made fewer errors during the first week of testing (Fig. 5g), and females spent more time in the maze (Fig. 5d) compared to their respective controls. The effect in females was both seen overall and during the first week of testing. Unfortunately, conclusions regarding sexual dimorphic effects on learning and memory cannot be drawn due to the different ages at which females and males were assessed (4–5 vs 8–9 months).

## Discussion

### PFHxS reduced thyroid hormone levels in rats without activation of the HPT axis

PFHxS exposure caused marked reductions in total T4^36^ and moderate reductions in T3 in both dams and offspring. Significant dose-dependent declines were evident in dams within 7 days, increasing in magnitude with continued exposures.

Despite the marked decreases in circulating thyroid hormone levels, no significant effects on TSH levels were found in dams (PD 22) or in offspring (PD 16 and PD 17). Based on the classic models of thyroid physiology and regulatory feedback loops, diminished levels of circulating T4 are accompanied by increases in TSH^2,45^. Thyrotropin-releasing hormone (TRH) activates the pituitary to release TSH, which binds to TSH receptors on the thyroid gland, upregulating synthesis and release of thyroid hormone into the blood. Indications of TSH-mediated thyroid gland activation include increases in thyroid gland weight, histological changes, and upregulation of certain gene transcripts^2,46–48^. The pattern of effects induced by the anti-thyroid drugs MMI and PTU conform to this classic view of the hypothalamic-pituitary-thyroid (HPT) axis. There are however a number of reports showing how PCBs, PBDEs and other PFASs exhibit another pattern of effects, corresponding to the one seen in the present study, i.e. clear reductions in circulating levels of T4 and T3 without an accompanying rise in TSH and activation of the HPT axis^14,27,49–53^. Consistent, with observations with another PFAS chemical, PFOS, reductions in serum T4 levels without HPT axis activation have been seen after both long-term adult exposure^50^ and developmental exposure of dams and pups^54,55^.

A previous report investigating PFHxS found histopathological changes in thyroid gland of adult male rats after 42 days of PFHxS exposure at 3 and 10 mg/kg, suggestive of HPT axis activation, but with no effects in dams or offspring following developmental exposures^35^. Unfortunately, TSH levels and thyroid gland weights were not reported^35^. Butenhoff *et al*. (2009) also reported clear sex differences in toxicokinetics, where parental females exposed to 3 and 10 mg/kg/day exhibited low serum PFHxS concentrations. In contrast, parental males had higher serum PFHxS levels, a different PFHxS distribution to organs, and showed clear signs of toxicity. The sex difference in adults is attributed to differential high expression of organic anion transporters in the kidneys of female rats that ensure efficient excretion of PFHxS into the urine^56–58^. Hence, male rats are exposed to much higher serum PFHxS levels than females. In agreement with our findings in rats, PFHxS showed no effect on TSH, thyroid gland weights nor histopathology in mouse dams and pups after exposure to somewhat lower internal PFHxS levels^37^. Unfortunately, T3 and T4 were not measured by Chang *et al*.^37^.

Based on the results on PFHxS exposed male rats^35^, although the long-term and high dose effects of PFHxS remain uncertain, it is clear that this class of chemicals does not activate the HPT axis in a manner similar to PTU/MMI, countering our understanding of HPT axis function. Despite a significant number of studies^49,51,53,59–61^, the mechanistic understanding of the phenomenon of marked reductions in circulating total T4 levels without a compensatory response within the HPT axis have yet to be determined, and the consequences of this form of thyroid hormone perturbation on brain development and function requires further study.

### Reduction in TH and mode of action

The mode of action (MoA) by which PFAS in general, and PFHxS specifically reduces serum T4 has not been fully elucidated, but the predominant knowledge revealed by *in vitro* assays suggest that these compounds bind to the serum distributor proteins TTR and albumin^62–64^. This MoA has been explored *in vivo* for the structurally similar perfluorinated compound PFOS (having a C-8 chain instead of the C-6 chain in PFHxS). Chang *et al*. (2008) showed a dramatic and transient rise in free serum T4 in the hours following administration of PFOS (presumably due to displacement of T4 from distributor proteins), followed by an increased turnover and loss of thyroid hormone through urine and feces^65^. Consistent with our findings, total T4 remained low without the expected rise in TSH. Here, we hypothesize that prolonged PFHxS exposure causes a steady-state T4 level at 30% of control levels with equilibrium between distributor-protein bound T4 and distributor-protein bound PFHxS.

Another possible thyroid MoA of PFAS is induction of liver enzymes (as a separate mode of action or secondary to TTR binding). PFOS exposure for 91 days decreased T4 levels, presumably through increased thyroidal conversion of T4 to T3 by deiodinase type 1 (increased Dio1 mRNA was reported) and increased degradation of T4 in liver by induction of thyroid hormone glucuronidation via liver enzyme UGT1A1^50^. However, the effects in the thyroid and liver were only evident at 5 mg PFOS/L drinking water and above, whereas clear effects on serum T4 were already detectable at the lower dose of 1.7 mg/L^50^. This disparity in dose level suggests that MoAs other than metabolism could underlie decrements in serum hormones. In our study, we did not observe an increase in *Dio1* expression in thyroid glands, whereas liver deiodinases and glucuronidases were not investigated.

Perfluorinated compounds may also augment hormone clearance through other pathways in the liver. Upregulation of the hepatic nuclear receptors peroxisome proliferator-activated receptor alpha (PPARα), constitutive androstane receptor (CAR) and pregnane X receptor (PXR) has been reported in PFHxS-exposed mice^37^ and PFOS-exposed rats^66^. With regard to other potential sites of chemical interference with thyroid hormone synthesis and signaling, PFHxS was a positive inhibitor of NIS *in vitro*^67^ but had no or minor activity at the thyroid receptor level^68,69^. Similarly, PFOS did not affect pituitary function in rats^65^. To our knowledge, the effects of PFHxS on thyroperoxidase (TPO) inhibition or peripheral deiodinases have yet to be investigated.

### PFHxS induced hypothyroxinemia and effects on the developing brain

While PFHxS caused dose-dependent reductions in serum T4 in both dams and offspring, these reductions were not associated with changes in cortical gene expression or any neurobehavioral effects measured in offspring.

#### Cortical gene expression

Targeted gene expression profiling was used to interrogate potential effects of thyroid hormone disruption on the developing rat neocortex. Based on a developmental PTU model, O’Shaughnessy *et al*., 2018 identified a suite of thyroid hormone responsive genes in neonatal cortex and suggested that they may serve as potential molecular readouts of TH action in the developing brain^40^. In this and other reports, expression levels of these genes were altered in a dose-dependent manner under conditions of thyroid hormone insufficiency^40,70,71^ or T3 activation^42,72^. On PD 16, where PFHxS-exposed pups exhibited reduced serum T4 levels, we hypothesized correlations between serum and brain T4 levels^26,40,73–76^, and conversely decreased thyroid hormone-mediated gene expression in the cortex. This however, was not the case. There are several possible explanations for this result. In PFHxS pups, T4 levels were reduced by 45%, a reduction similar to the effect size necessary to observe effects on brain T3, T4 and brain gene expression in a similar study with the potent anti-thyroid drug PTU (obtained at an exposure of 1 ppm PTU on PD 14)^40^. Therefore, the 45% reduction in serum T4 by PFHxS may have been at the threshold for imparting a molecular signal in the brain. After PTU exposure at the lowest dose of 1 ppm, the reduction in gene expression was modest but its significance strengthened by the severe and unambiguous reductions at higher doses^40^. As PFHxS is less potent than PTU in terms of the maximal degree of serum T4 reduction obtained, the modest effects by PFHxS exposure on only two of the 8 cortex genes remain equivocal. Additionally, severe and sustained reductions in serum/brain thyroid hormone inclusive of fetal and earlier postnatal periods that disrupt development trajectories may be necessary to provide a foundation for the gene expression changes in cortex observed with PTU. Comparable degrees of hormone perturbation may not have been achieved by PFHxS during critical fetal and early neonatal time windows. The findings suggest that snapshots of serum T4 levels, although important indicators of the potential of thyroid mediated neurotoxicity, may not serve as direct surrogates for brain hormone concentrations and consequent thyroid action under all xenobiotic exposure conditions. Compensatory mechanisms in the PD 16 pup brain, including the upregulation of local deiodinases and thyroid hormone transporters, may be sufficient in PFHxS exposed pups to maintain local levels of brain hormones and support normal transcription of cortical gene targets investigated in this study. Alternatively, genes other than the ones examined here or in different brain regions and at different developmental times may have been affected by PFHxS exposure.

#### Functional tests of brain development following hypothyroxinemia

PFHxS exposure did not alter motor activity assessed at three time-points in the post-weanling rat or impair performance in the radial arm maze. Does this suggest that PFHxS is without effect on brain development? Perhaps, but it may be the case that the current metrics applied in rodent models are not sufficiently sensitive to detect adverse neurodevelopmental effects of maternal and perinatal hypothyroxinemia.

We chose the behavioral tests based on previous work using PTU to disrupt thyroid action, in which functional impairments were observed in adult offspring despite full recovery of thyroid hormone status^13^. In models where severe hormone deficiencies are induced by PTU, MMI or a thyroidectomy, there is a clear association between reductions in serum T4 during the perinatal period and a wide range of behavioral impairments^13–20^. In contrast, studies investigating developmental hypothyroxinemia induced by other substances, have not shown behavioral effects clearly correlated to T4^22–27^. We hypothesize that some of the discrepancy between PTU and environmental chemicals arises from differences in timing, severity and duration of the induced fetal and postnatal T4 suppression. These factors are highly influenced by toxicokinetics, complicated under conditions of pregnancy and lactation, and likely MoA of chemical interference with thyroid signaling. The data on fetal and brain T4 supply indicate that PTU very effectively restricts fetal serum and brain T4^40,70^ while not much is known for most other compounds. Possibly, rodent behavior models detect effects after only very marked and sustained reductions in fetal and neonatal thyroid hormones as seen in studies where the lowest doses of PTU did not induce significant deficits in neurobehavior^13,14,17,20,77^. Yet, there was impaired synaptic plasticity and structural defects in the forebrain^19,20^ indicating that neurotoxicity can exist despite the lack of behavioral readout, supporting the view that simple behavioral tests typically employed in rodent toxicology studies lack sensitivity to detect consequences from modest serum T4 reductions. Still, in humans, adverse effects on neurobehavioral indices are found in children of women within low-normal range of circulating serum T4^7,9–11,78,79^ indicating that chemically-induced thyroid hormone disruption during pregnancy is a potential risk to human brain development. Functional parameters such as IQ and language development are commonly affected, but these behavioral domains are not readily assessed in rodent models. Documented ‘functional’ impairment however is a critical component of the regulatory framework within the EU. Identification of an endocrine disrupting chemical in the EU is based on the World Health Organization (WHO) definition: *an exogenous substance that alters the function of the endocrine system to cause adverse health effects in an intact organism or its progeny*^3^. For regulatory actions to be taken on thyroid disrupting chemicals in the EU system, evidence of reduction in circulating hormone *must* be associated with an adverse (neurodevelopmental) effect and be plausibly linked to a MoA relevant to the thyroid system. Therefore, there is a critical need to develop reliable and sensitive assays to detect neurological impairments associated with developmental thyroid hormone disruption.

### Effects by low doses and on sexual differentiation of the brain

Despite the relative absence of thyroid hormone-dependent effects of PFHxS on functional outcomes in this study, we did discover effects of the lowest dose of PFHxS suggestive of altered sexual differentiation of the brain, and on learning and memory. These effects were restricted to the lowest dose of 0.05 mg/kg PFHxS and were uncoupled from the serum thyroid hormone reductions evident at higher doses. The EDmix itself significantly increased activity in both PD 27 and PD 115 males, i.e. making these males more “female-like” in their behavior, supporting our previous studies^36^, where weak signs of sex hormone disruption (nipple retention and alterations in male reproductive tissues) were induced by the EDmix and 0.05 mg/kg PFHxS. The subtle and non-monotonic nature of the response raises the possibility that these observations could be the result of sampling error. However, if valid, effects at such a low dose are of heightened concern for human health and warrant further study. The effects occurred at a dose far lower than what would be tested in traditional developmental toxicology studies and consequently could be missed in standard regulatory testing scenarios. Additional studies targeting the dose-response relationships for effects on sex differentiation and behavior could serve to expand our understanding of these elusive effects and improve our ability to safeguard human health in the future.

## Conclusion

PFHxS reduced T3 and T4 in pregnant dams and their progeny but did not appear to activate the HPT axis at doses up to 25 mg/kg bw/day. The thyroid hormone disruptions were not correlated with effects on motor activity or learning and memory; rather the findings suggest that the primary effect of low doses of PFHxS may be to disrupt sexual differentiation of the brain.

Human data show that T4 reductions during pregnancy and in newborns can impair brain development and cause reductions in a child’s IQ. Despite PFHxS-induced reductions in circulating T4 levels, no evidence of thyroid hormone-mediated neurobehavioral disruption in offspring was observed. However, we maintain that a significant reduction in T4 alone should warrant concern and that the metrics currently applied in rodent models are not sufficiently sensitive to detect adverse neurodevelopmental effects of maternal and perinatal hypothyroxinemia. But until we have discovered more sensitive brain-based markers or measurable metrics of thyroid hormone-dependent perturbations in brain development, we cannot with certainty determine whether or not PFHxS-mediated reductions in circulating thyroid hormone levels can adversely affect brain development.

## Materials and Methods

### Animals and treatment

The full animal study design has been previously reported^36^, and herein depicted in Fig. 1. Briefly, 144 time-mated Wistar rats were divided across 4 balanced blocks and into 8 groups of 16–20 animals each. PFHxS (denoted “Px” in group names, Sigma-Aldrich, tridecafluorohexane-1-sulfonic acid potassium salt, purity > 98%, CAS-No: 3871-99-6, lot #BCBC3545V) doses were 0.05, 5 and 25 mg/kg body weight (bw)/day, given both with and without a fixed dose of background EDmix (denoted “+ED” in group names) exposure, a vehicle control group, and a group receiving only the EDmix. Corn oil (Sigma-Aldrich) was used as both vehicle and as a control compound. The EDmix was made up of 12 endocrine disrupting chemicals (EDmix) comprising 6 pesticides: vinclozolin (0.9 mg/kg bw/day), prochloraz (1.4 mg/kg bw/day), procymidone (1.5 mg/kg bw/day), linuron (0.06 mg/kg bw/day), epoxyconazole (1 mg/kg bw/day), and dichlorodiphenyldichloroethylene (4,4′-DDE) (0.1 mg/kg bw/day), 2 UV-filters: 4-methylbenzylidene camphor (6 mg/kg bw/day) and octyl methoxycinnamate (12 mg/kg bw/day), 3 plasticizers: dibutyl phthalate (1 mg/kg bw/day), di-2-ethylhexyl phthalate (2 mg/kg bw/day) and bisphenol A (0.15 mg/kg bw/day), and 1 preservative: butyl paraben (6 mg/kg bw/day). The EDmix composition and exposure level reflected 100 times high-end human intakes as has been described previously^38,39^. However, acetaminophen (paracetamol) that was included in the prior study by Christiansen *et al*. (2012), was not included in the EDmix of this study. The resulting total EDmix dose was 32.11 mg/kg bw/day^36^. The EDmix contained some chemicals capable of disrupting thyroid hormone levels, although given at doses well below reported No Observed Adverse Effect Levels (NOAELs). Combined in a mixture, however, these chemicals may have contributed to the observed effect by the EDmix on dam T4 levels^36^.

Pregnant dams were received on gestation day (GD) 3 of pregnancy (the day following overnight mating was designated GD 1) and dams were dosed once daily by oral gavage from GD 7 to postnatal day (PD) 22, but not on the day of delivery. The expected day of delivery (GD 23) was termed PD 1 for all pups. Hence, the age of the pups was related to time of mating rather than day of birth. Animals were housed in a controlled environment: Reversed light/dark cycles of 12 hours (light from 9 pm–9 am), humidity 55 ± 5%, temperature at 21 ± 1 °C, and ventilation changing air ten times per hour. All animals were fed ad libitum on a standard diet with Altromin 1314 (soy and alfalfa-free with an iodine content of 1.52 mg/kg, Altromin GmbH, Lage, Germany) and were provided ad libitum acidified tap water (to prevent microbial growth) in PSU bottles (84-ACBTO702SU Tecniplast).

At weaning (PD 22), one male and one female pup (when available) from each litter were weaned and housed pairwise with an animal of the same sex and group (with a sibling if another animal of same sex and group was not available). This cohort of weaned offspring was subjected to behavioral assessment of motor activity levels at three ages, and for the control and PFHxS-only groups, learning and memory were tested in the radial arm maze (see below for descriptions). The study was terminated when the males were approximately 12 months and the females 13 months old.

The animal experiments were carried out at the DTU National Food Institute facilities (Mørkhøj, Denmark). Ethical approval was given by the Danish Animal Experiments Inspectorate. The authorization number given is 2015-15-0201-00553 C3. The experiments were overseen by the National Food Institute’s in-house Animal Welfare Committee for animal care and use. All methods in this study were performed in accordance with relevant guidelines and regulations.

### Autopsies

Necropsy of one male and one female (when available) per litter took place on PD 16 and PD 17, respectively, and again on PD 22, when dams were also included (see below).

#### Dams

On PD 22 dams were weighed, anesthetized with CO_2_/O_2_, decapitated, and trunk blood collected for hormone analysis (see below). Livers were excised and saved for histopathological examination (control and 25-Px groups)(see below). Thyroid glands were excised, weighed and saved for histopathology (control and PFHxS-only groups)(see below) in block 1–3. Thyroid glands from block 4 were stored in RNAlater at −80 °C for analysis of gene transcripts (control and PFHxS-only groups).

#### Offspring

On PD 16/17 and PD 22 pups were weighed, anesthetized with CO_2_/O_2_, decapitated, and trunk blood collected for hormone analysis. Livers from females were excised and saved for histopathology (PD 17)(see below). Male pup thyroid glands were excised with a piece of the thyroid cartilage and saved for histopathology (see below). Thyroid glands from females were excised, weighed, and those from block 1 and 2 were stored in RNAlater at −80 °C for gene transcript analysis (control and PFHxS-only groups).

### Thyroid hormones and thyroid stimulating hormone (TSH)

On GD 15, tongue blood was taken from the dams without anesthesia. Male pups on PD 16 and female pups on PD 17 were euthanized by decapitation and trunk blood collected. Dam trunk blood was also collected at weaning of the pups on PD 22. The blood was collected in heparinized microcentrifuge tubes (GD 15) or in heparinized 4- or 10 ml vacutainer tubes for the pup blood and the dam blood, respectively. Blood was kept on ice until centrifugation for 10 min. at 4 °C and 4000 rounds per minute (rpm) and plasma was stored at −80 °C. T3 levels were analyzed by electrochemiluminescence-immunoassay (ECLIA) – photoncount (Cobas 8000 E-modul) at the Department of Clinical Biochemistry, Rigshospitalet, Copenhagen, Denmark. For the dams, 15 samples from each exposure group (20 controls) were sampled randomly for analysis. For the pups, each litter was represented by either a male or female pup (the different sampling times for male and female pups limit conclusions on sex-specific effects).

Thyroid Stimulating Hormone (TSH) was measured in rat plasma with the Milliplex MAP Rat Thyroid Magnetic Bead Panel Luminex (RTHYMAG-30K, Merck Millipore) according to the instructions of the manufacturer on randomly chosen samples from male (n = 11–13) and female pups (n = 5–7) PD 16/17 (control, 5-Px and 25-Px groups only) and dams PD 22 (control and PFHxS-groups only).

### Liver and thyroid gland histopathology

#### Liver

A predefined (thin and uniform) standardized slice of the liver from the left lateral and right medial lobes were fixed in 10% formalin, processed, embedded in paraffin, and 5μm sections were stained with haematoxylin and eosin (H&E) following standard procedures. Livers from dams (PD 22) and female offspring (PD 17) were evaluated, blinded to exposure groups, for hepatocellular hypertrophy and vacuolation (macro- and microvesicular) in the control and high-dose (25-Px) groups.

#### Thyroid gland

Sections of thyroid glands from dams (PD 22) (control, 25-Px, EDmix, and 25-Px + ED groups) and male offspring (PD 16) (control and 25-Px groups) were fixed in 10% formalin, embedded in paraffin, and 5 μm sections were stained with H&E. Histological examination of thyroid glands was conducted blinded to exposure groups to evaluate signs of pathological changes as detailed below.

Thyroid glands from dams and male pups were assigned to one of the following three categories:

- A: No remarks
- B: Mild alterations with few irregular follicles or few follicles with multi-layered epithelium, increased cellularity.
- C: Follicular epithelial hypertrophy or hyperplasia, irregular cells or follicles, multi-layered follicular epithelium, papillary projections in follicular lumens and/or small peripheral follicles localized on the rim of the gland.

### Thyroid gland and cerebral cortical gene expression by RT-qPCR

Both lobes of the thyroid gland from female PD 17 offspring were stored in RNAlater (Ambion AM7021) at −80 °C. until RNA extraction. Thyroid glands from PD 22 dams were snap frozen in liquid nitrogen and stored at −80 °C until analysis of one lobe. An oblique slab of anterior to lateral cortex was collected from PD 16 male offspring and stored in RNAlater at −80 °C until RNA extraction. RNA was extracted with TRI Reagent (Sigma T9424) according to manufacturer’s protocol and glycogen added to facilitate precipitation of the thyroid gland RNA. RNA pellets were resuspended in nuclease-free water, DNased (RQ1 DNase, Promega M6101) and quantitated with Quant-iT RiboGreen RNA assay kit (Life Technologies R11490e). 2 µg RNA was reverse transcribed (Life Technologies High Capacity cDNA Reverse Transcription Kit 4374966) and the resulting cDNA was amplified using TaqMan Gene Expression Assays (Life Technologies) and TaqMan Gene Expression PCR Master Mix (Life Technologies 4369510) according to manufacturer’s protocol. Amplification was performed on an ABI model 7900HT sequence detection system in duplicates. TaqMan probes are detailed in Table 1. Transcripts evaluated in thyroid glands were: *Slc5a5* (NIS), *Nkx2.1, Tpo*, *Tshr*, *Pax8*, and *Dio1*. Transcripts evaluated in cortical samples included: *Agt*, *Col11a2*, *Gjb6*, *Hr*, *Itih3*, *Klf9, Ntf3*, and *Pvalb*. *B2m* and *Gapdh* were used as reference genes for thyroid gland and cerebral cortex, respectively. Data were analyzed by the 2^−∆∆Ct^ method^80^.

**Table 1:** List of genes and corresponding TaqMan assays.

Gene	RefSeq	Name	TaqMan
**Thyroid gland**			
*B2m*	NM_012512	Beta-2-microglobulin	Rn00560865
*Dio1*	NM_021653	iodothyronine deiodinase type 1	Rn00572183
N*kx2.1*	NM_013093	NK2 homeobox 1 (TTF-1)	Rn01512482
*Pax8*	NM_031141	Paired box 8	Rn00579743
*Slc5a5* (NIS)	NM_052983	Solute carrier family 5	Rn00583900
*Tpo*	NM_019353	Thyroid peroxidase	Rn00571159
*Tshr*	NM_012888	Thyroid stimulating hormone receptor	Rn00563612
**Cortex**			
*Agt*	NM_134432	Angiotensinogen	Rn00593114
*Col11a2*	NM_212528	Collagen type XI alpha 2 chain	Rn01428773
*Gapdh*	NM_017008	glyceraldehyde-3-phosphate dehydrogenase	Rn01775763
*Gjb6*	NM_053388	Gap junction beta 6	Rn02042582
*Hr*	NM_024364	Hairless	Rn00577605
*Itih3*	NM_017351	Inter-Alpha-Trypsin Inhibitor Heavy Chain 3	Rn00569293
*Klf9*	NM_057211	Kruppel like factor 9	Rn00589498
*Ntf3*	NM_031073	Neurotrophin-3	Rn00579280
*Pvalb*	NM_022499	Parvalbumin (parv)	Rn00574541

### Brain function

#### Motor activity and habituation in offspring at 3 ages

One male and one female pup from each litter were assessed for motor activity on PD 27. The same animals were tested as young adults (PD 115) and again later in life (PD 342/336).

The animals were placed individually in clean, empty cages with a flat rack lid. Cages were placed in activity boxes with photocells recording horizontal activity for 30 min. Six animals were tested simultaneously and, in all test rounds, both male and female offspring were represented. Neither food nor water were supplied during the testing. Movement was registered as interruption of a photobeam and data automatically collected by a computer in an adjoining room. The data collection started after a 10 second habituation and continued for 10 periods of 3 minutes amounting to a total of 30 minutes of observation. Motor activity was quantified as activity counts (disruption of adjacent photobeams) and break counts (total number of interrupted photobeams). Activity counts allowed determination of whether the animal was moving about the cage and not just continuously interrupting the same beam. Total activity during the 30 minutes was used as a measure of general activity and compared across groups for each sex as well as within groups for sex difference. To assess habituation, activity counts were divided into three periods, initial (1–9 min), middle (10–21 min), and last period (22–30 min).

#### Radial arm maze

Adult offspring from the PFHxS-only groups (0, 0.05, 5, and 25 mg/kg/day PFHxS) were tested in the radial arm maze. One male and one female from each litter were assessed, females at 4–5 months and males at 8–9 months of age. This age difference was necessitated by the large number of animals to be tested and the limitations of the capacity of the maze system. All animals had previously been tested on motor activity. Due to technical difficulties, the radial arm maze was reduced to 7 arms. In the pre-testing week the animals were fed only 15 g of chow a day in the afternoon and hand-fed a raw peanut (Brogaarden, Denmark) to accustom them to the taste of the food reward used in the maze. Following the pre-testing week, the animals received 1 trial/day in the radial arm maze. Animals were introduced to the central space of the maze and allowed to explore until they had entered and traversed to the end of each arm or until 10 mins had elapsed. Testing was performed for five consecutive days, followed by a 2-day break for a total of three weeks. Analysis was performed based on calculations of “time” (time to visit all arms) and errors (number of visits to arms already visited, in addition to number of arms not visited on a given day). Data analysis included measures of total time and errors for each animal. To assess learning over time a mean per day during week 1, 2 and 3 was calculated.

### Statistical analysis

Data with normal distribution and homogeneity of variance were analyzed using analysis of variance (ANOVA). Data were transformed if normal distribution and homogeneity of variance was not present. When relevant, body weight was included as a covariate in the analyses, e.g. when testing terminal organ weights. Histology results were analyzed with Fisher’s test when analyzing 2 groups and Chi square tests for more than two groups (i.e., dam thyroid glands). To avoid litter effects no more than one pup per litter was included in any analysis. Where an overall significant treatment effect was observed, Dunnett’s test was used for two-tailed comparison with the alpha level for statistical significance always set to 5%. Exposure groups were tested against the vehicle control by testing the PFHxS exposed groups against the control, all PFHxS + EDmix groups against the EDmix group and by using a t-test to compare the EDmix group against the control group. In addition, the study design with dose response exposure to PFHxS and the addition of groups exposed to both PFHxS and the EDmix allowed for construction of a statistical model integrating all exposures and control groups into one full linear model the “full model” (described in detail in Ramhøj *et al*., 2018). The full model included the PFHxS and EDmix exposures with their interactions parameterized as indicator variables. This supported a determination as to whether (and to what magnitude) exposure to the EDmix changed the effects of PFHxS exposure alone across the different exposure groups. Because many samples were considered in the full model, much smaller effect changes could be identified than would be possible using a simple pairwise comparison. The model was tested by a main factor for EDmix and dose-dependent interaction terms to account for non-parallel dose-response patterns between PFHxS and PFHxS + EDmix.

SAS Enterprise Guide 4.3 (2010), SAS Institute Inc, Cary, NC 27513, USA, was used for statistical analyses.

## Supplementary information

Supplementary Information.
