# Supplementary material for: Evaluating thyroid hormone disruption: investigations of long-term neurodevelopmental effects in rats after perinatal exposure to perfluorohexane sulfonate (PFHxS)
Source: Sci Rep. 2020 Feb 14;10:2672. doi: 10.1038/s41598-020-59354-z (PMC7021709; doi:10.1038/s41598-020-59354-z)
Supplement: Supplementary file 1 — Supplementary Information. [file 41598_2020_59354_MOESM1_ESM.docx]

**Evaluating thyroid hormone disruption: investigations of
long-term neurodevelopmental effects in rats after perinatal exposure to perfluorohexane sulfonate (PFHxS)**

Louise Ramhøj, Ulla Hass, Mary Gilbert, Carmen Wood, Terje Svingen, Diana Usai,
Anne Marie Vinggaard, Karen Mandrup & Marta Axelstad

Supplementary information


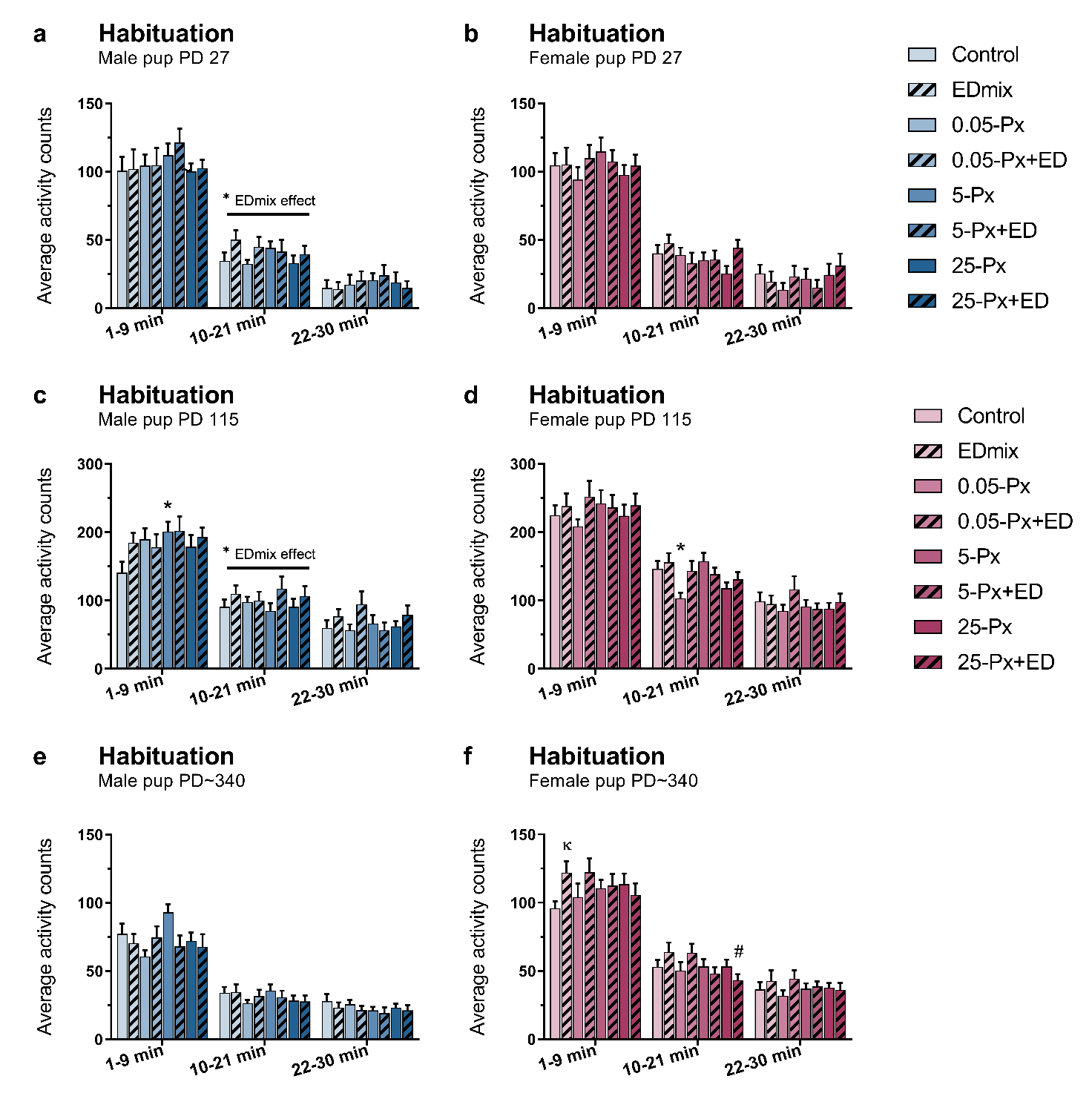


**Supplementary Figure 1. Minor effects on activity levels and habituation in young (PD 27), adult (PD 115) and old offspring (PD ~340) exposed to PFHxS with or without EDmix during development**. The motor activity levels were assessed in a new environment over the course of 30 min and divided into three time periods to assess habituation patterns: first (1-9 min), middle (10-21 min) and last (22-30 min) test periods (total activity counts are shown in Fig. 5). **(a)** EDmix increased activity in the middle test period in the 27 day old male offspring (p = 0.0414, full model). **(b)** No effects in female PD 27 offspring. **(c)** The EDmix decreased habituation in the adult male offspring on PD 115. Specifically there were statistically significantly increased activity levels in the middle and last test period (p = 0.0437 and p = 0.0297, respectively, full model). PFHxS at a dose of 5 mg/kg caused increased initial activity on PD 115 (p = 0.018 compared to control in a simple statistical model and p = 0.0289 in the full model). **(d)** In the adult female offspring the lowest dose of PFHxS (0.05 mg/kg) decreased activity in the middle test period (p = 0.014 compared to control in a simple statistical model and p = 0.0337 in the full model). **(e)** No statistically significant effects were found in male offspring on PD ~340. **(f)** EDmix increased activity levels, compared to the control group, in the first test period in old female offspring PD ~340 (p = 0.0082 in a t-test comparing the EDmix group to the control group). The highest dose of PFHxS in combination with the EDmix decreased activity levels in the middle test period as compared to the EDmix only group (p = 0.034 compared to the EDmix).
Pink bars represent female offspring and blue bars male offspring. n = 15-17 (except for EDmix, 0.05-Px+ED and 5-Px+ED with n = 12-13) animals per sex per litter. Data shown as mean + SEM, * p <0.05 compared to control, # p <0.05 compared to EDmix, κ p<0.05 in a t-test comparing the EDmix group to the control. ED: EDmix. Px: PFHxS. PD: postnatal day.
